# Synthesis and Biological Evaluation of Thiophene-Based Cannabinoid Receptor Type 2 Radiotracers for PET Imaging

**DOI:** 10.3389/fnins.2016.00350

**Published:** 2016-07-27

**Authors:** Achi Haider, Adrienne Müller Herde, Roger Slavik, Markus Weber, Claudia Mugnaini, Alessia Ligresti, Roger Schibli, Linjing Mu, Simon Mensah Ametamey

**Affiliations:** ^1^Department of Chemistry and Applied Biology, Institute of Pharmaceutical Sciences, Swiss Federal Institute of Technology Zurich, Switzerland; ^2^Department of Molecular and Medical Pharmacology, University of California, Los Angeles Los Angeles, CA, USA; ^3^Neuromuscular Diseases Unit/ALS Clinic, Kantonsspital St. Gallen St. Gallen, Switzerland; ^4^Department of Biotechnology Chemistry and Pharmacy, University of Siena Siena, Italy; ^5^Institute of Biomolecular Chemistry, National Research Counsil of Italy Naples, Italy; ^6^Department of Nuclear Medicine, University Hospital Zurich Zurich, Switzerland

**Keywords:** cannabinoid receptor type 2, neuroinflammation, positron emission tomography, neurodegenerative disorders, thiophene-based structures

## Abstract

Over the past two decades, our understanding of the endocannabinoid system has greatly improved due to the wealth of results obtained from exploratory studies. Currently, two cannabinoid receptor subtypes have been well-characterized. The cannabinoid receptor type 1 (CB_1_) is widely expressed in the central nervous system, while the levels of the cannabinoid receptor type 2 (CB_2_) in the brain and spinal cord of healthy individuals are relatively low. However, recent studies demonstrated a CB_2_ upregulation on activated microglia upon neuroinflammation, an indicator of neurodegeneration. Our research group aims to develop a suitable positron emission tomography (PET) tracer to visualize the CB_2_ receptor in patients suffering from neurodegenerative diseases. Herein we report two novel thiophene-based ^11^C-labeled PET ligands designated [^11^C]AAT-015 and [^11^C]AAT-778. The reference compounds were synthesized using Gewald reaction conditions to obtain the aminothiophene intermediates, followed by amide formation. Saponification of the esters provided their corresponding precursors. Binding affinity studies revealed K_i_-values of 3.3 ± 0.5 nM (CB_2_) and 1.0 ± 0.2 μM (CB_1_) for AAT-015. AAT-778 showed similar K_i_-values of 4.3 ± 0.7 nM (CB_2_) and 1.1 ± 0.1 μM (CB_1_). Radiosynthesis was carried out under basic conditions using [^11^C]iodomethane as methylating agent. After semi-preparative HPLC purification both radiolabeled compounds were obtained in 99% radiochemical purity and the radiochemical yields ranged from 12 to 37%. Specific activity was between 96 and 449 GBq/μmol for both tracers. In order to demonstrate CB_2_ specificity of [^11^C]AAT-015 and [^11^C]AAT-778, we carried out autoradiography studies using CB_2_-positive mouse/rat spleen tissues. The obtained results revealed unspecific binding in spleen tissue that was not blocked by an excess of CB_2_-specific ligand GW402833. For *in vivo* analysis, [^11^C]AAT-015 was administered to healthy rats via tail-vein injection. Evaluation of the CB_2_-positive spleen, however, showed no accumulation of the radiotracer. Despite the promising *in vitro* binding affinities, specific binding of [^11^C]AAT-015, and [^11^C]AAT-778 could not be demonstrated.

## Introduction

Cannabinoid receptors belong to the large family of G protein-coupled receptors (GPCRs) exhibiting the characteristics of a glycosylated extracellular N-terminus, seven transmembrane α-helixes, and an intracellular C-terminus (Mackie, 2008; Rom and Persidsky, 2013). They are key players in a series of physiological processes resulting in an overall auto-protective effect in mammalians (Pertwee, 2005). Currently, there are two well-characterized subtypes of the cannabinoid receptor (CB_1_ and CB_2_) that share 44% sequence similarity and differ substantially in their expression profiles (Pertwee, 1997). The CB_1_ receptor is mainly expressed in the central nervous system (CNS), CB_2_ is predominantly found in peripheral immune cells (Matsuda et al., 1990; Herkenham et al., 1991; Maresz et al., 2005; Chin et al., 2008). Under physiological conditions, the CB_2_ receptor expression in the brain and the spinal cord is barely detectable (Van Sickle et al., 2005). However, there are several studies demonstrating an upregulation of CB_2_ on activated microglial cells (macrophages of the CNS), rendering the receptor a promising target to exploit neuroinflammatory changes involved in neurodegenerative disorders such as multiple sclerosis, amyotrophic lateral sclerosis (ALS), Parkinson's or Alzheimer's disease (Benito et al., 2008; Onaivi, 2009).

The various implications of the CB_2_ receptor as well as the fact that it is only marginally expressed in the CNS of healthy subjects make it a promising target for diagnostic and clinical applications. The development of a successful CB_2_ PET ligand might improve our understanding of the mechanisms underlying disease pathogenesis as well as provide a tool for therapy monitoring and development of novel therapeutic drug candidates. In particular, this would be beneficial for patients suffering from rare diseases with no current effective drug treatment like ALS (Zinman and Cudkowicz, 2011). Several CB_2_ radioligands have been published by various research groups but till now, none of these radioligands has been found to be useful in the clinics. Their limitations included lack of selectivity over CB_1_, high lipophilicity, low brain uptake due to P-glycoprotein efflux, brain penetrating radiometabolites, and trapped radiometabolites (Evens et al., 2008; Horti et al., 2010; Vandeputte et al., 2011; Hortala et al., 2014). Our research group is interested in evaluating different structural scaffolds in order to determine potential candidate compounds as CB_2_-specific radioligands. In our previous work, we reported the development of oxoquinoline- as well as pyridine-based CB_2_ radiotracers (Mu et al., 2013, 2014; Slavik et al., 2015a,b). Recently, a new class of thiophene amide derivatives emerged as potent and selective CB_2_ agonists (Nelson et al., 2012). Several studies have been conducted to show the therapeutic applicability of thiophene-based derivatives in pain management, but this class of compounds has not yet been tested within the context of neurodegeneration. Herein we report the development of two novel thiophene-based PET radioligands designated [^11^C]AAT-015 and [^11^C]AAT-778 for a diagnostic approach toward CB_2_ receptor imaging.

## Materials and methods

All animal experiments were carried out in accordance with the Swiss Animal Welfare legislation and approved by the Veterinary Office of the Canton Zurich. Male Wistar rats and male CD1 mice were purchased from Charles River Laboratories (Sulzfeld, DE) and kept under standard conditions.

All chemicals, unless otherwise stated, were purchased from Sigma Aldrich GmbH (Taufkirchen, DE), ABCR GmbH (Karlsruhe, DE), Merck (Darmstadt, DE), or Fluka (Buchs, CH) and were used without further purification. Solvents for thin layer chromatography (TLC), column chromatography and extractions were purchased as commercial grade. Organic chemistry reactions were monitored by TLC using Sigma-Aldrich silica gel 60 plates under UV light at 254 nm. Nuclear magnetic resonance (NMR) spectra (^1^H and ^13^C NMR) were obtained on a Bruker Avance FT-NMR spectrometer (400 MHz). Chemical shifts are given in delta (δ) units, in ppm relative to tetramethylsilane (TMS, 0 ppm). Multiplicities in the ^1^H NMR spectra are described as: s = singlet, d = doublet, t = triplet, m = multiplet and br = broad peak. Coupling constants (J) are reported in Hz. High resolution mass spectrometry (HRMS) was performed on a Bruker's maXis (ESI-Qq-TOF-MS) spectrometer (Bruker Daltonik GmbH, DE) and are given in m/z.

High-performance liquid chromatography (HPLC) analysis were performed using a reversed phase column (ACE column, C18, 3 μm) with a gradient system of acetonitrile and 0.1% trifluoroacetic acid in water. Analytical radio-HPLC was performed with a flow rate of 1 mL/min on an Agilent 1100 series system equipped with a Raytest Gabi Star radiodetector (Agilent Technologies, Morges, CH). Semi-preparative HPLC purifications were carried out using a reversed phase column (ACE column, Symmetry C8 5 μm; 7.8 × 50 mm) under the following conditions: 0.1% H_3_PO_4_ in H_2_O (solvent A), MeCN (solvent B); 0.0–8.0 min, 20% B; 8.1–30.0 min, 20–90% B; 30.1–32.0 min, 90% B; 32.1–34.0 min, 90–20% B; 34.1–40.0 min, 20% B; flow rate: 4 mL/min. Specific activity was calculated by comparing ultraviolet peak intensity of the final formulated products with calibration curves of corresponding non-radioactive standards of known concentrations.

### Chemistry

#### Methyl 2-amino-5-methyl-4-propylthiophene-3-carboxylate (1)

Morpholine (0.87 mL, 10 mmol) was added to a mixture of 3-hexanone (1.0 g, 10 mmol), methyl cyanoacetate (0.88 mL, 10 mmol) and sulfur (0.32 g, 10 mmol) in MeOH (5 mL). The mixture was refluxed overnight, then cooled to room temperature and poured into ice water. The resulting precipitate was filtered and the solid was then dissolved in dichloromethane. The solution was dried over anhydrous sodium sulfate and evaporated to dryness. The residue was purified by flash column chromatography on silica gel using petroleum ether/ethyl acetate (9:1) as eluent to give the title compound (230 mg, 11%) as a white solid. ^1^H NMR (400 MHz, DMSO-*d*_6_): δ 7.14 (s, 2H), 3.68 (s, 3H), 3.32 (s, 3H), 2.54 (t, *J* = 7.4 Hz, 2H), 1.38 (m, 2H), 0.85 (t, *J* = 7.4 Hz, 3H). ^13^C NMR (100 MHz, DMSO-*d*_6_): δ 165.0, 162.4, 136.2, 112.5, 103.0, 50.3, 29.6, 23.2, 13.9, 12.0. HRMS calculated for C_10_H_16_NO_2_S (M+H) 214.0896, found 214.0897.

#### Methyl 2-(adamantane-1-carboxamido)-5-methyl-4-propylthiophene-3-carboxylate (AAT-778)

A solution of 1-adamantane-carbonyl chloride (240 mg, 1.22 mmol) in dry 1,4-dioxane (3 mL) was added dropwise to a solution of compound **1** (200 mg, 0.94 mmol) in 10 mL of the same solvent while maintaining the reaction temperature at 70°C. After the addition was completed, the solution was stirred at 100°C for 2 h and then concentrated under reduced pressure. The residue was dissolved in dichloromethane, washed with aq. HCl (1M) and NaHCO_3_ (saturated solution), dried over anhydrous sodium sulfate and evaporated to dryness. The crude product was purified by flash column chromatography on silica gel and eluted with petroleum ether/ethyl acetate (9:1). Subsequent recrystallization from MeOH gave the title compound (166 mg, 47%). ^1^H NMR (400 MHz, CDCl_3_): δ 11.6 (s, 1H), 3.9 (s, 3H), 2.7 (t, *J* = 6.7 Hz, 2H), 2.2 (s, 3H), 2.1 (m, 3H), 2.0 (m, 7H), 1.8 (m, 7H), 1.5 (m, 2H), 0.9 (t, *J* = 6.7 Hz, 2H). ^13^C NMR (100 MHz, CDCl_3_): δ 175.3, 167.1, 148.0, 133.7, 123.6, 111.6, 51.4, 39.0, 36.4, 29.8, 28.0, 23.8, 14.0, 12.3. HRMS calculated for C_21_H_30_NO_4_S (M+H) 392.1890, found 392.1888.

#### 2-(adamantane-1-carboxamido)-5-methyl-4-propylthiophene-3-carboxylic acid (2)

A solution of NaOH (140 mg, 3.5 mmol) in water (2 mL) was added to a solution of **AAT-778** (100 mg, 0.26 mmol) in MeOH (2 mL) and the mixture was stirred at 80°C for 3 h. After cooling, aq. HCl (1 M) was added until a pH of 2 was reached. The resulting precipitate was filtered and dried to afford the pure acid (71 mg, 75%) as a white solid. ^1^H NMR (400 MHz, DMSO-*d*_6_): δ 13.2 (s, 1H), 11.7 (s, 1H), 2.7 (t, *J* = 5.7 Hz, 2H), 2.2 (s, 3H), 2.0 (m, 3H), 1.9 (m, 7H), 1.7 (m, 7H), 1.4 (m, 2H), 0.9 (t, *J* = 6.7 Hz, 2H). ^13^C NMR (100 MHz, DMSO-*d*_6_): δ 174.5, 167.8 146.7, 134.3, 123.2, 112.7, 38.9, 36.3, 29.4, 27.9, 23.8, 14.3, 12.4. HRMS calculated for C_20_H_27_NO_3_S (M+H) 362.1784, found 362.1781.

#### Methyl 2-amino-4,5,6,7-tetrahydrobenzo[b]thiophene-3-carboxylate (3)

To a suspension of sulfur in MeOH (10 mL) was added methyl 2-cyanoacetate (0.93 mL, 10.54 mmol) and cyclohexanone (0.993 mL, 9.58 mmol). Subsequent addition of diethylamine (0.49 mL, 4.69 mmol) gave a clear solution which was stirred at 50°C for 12 h. After cooling to room temperature, the reaction mixture was poured into ice-water and the resulting precipitate was filtered. The crude product was purified by flash column chromatography on silica gel using hexane/ethyl acetate (8:2) as eluent to obtain compound **3** (1.570 g, 78%). ^1^H NMR (400 MHz, DMSO-*d*_6_): δ 7.20 (s, 2H), 3.66 (s, 3H), 2.57 (t, *J* = 6.0 Hz, 2H), 2.41 (t, *J* = 6.0 Hz, 2H), 1.66 (m, 4H). ^13^C NMR (100 MHz, DMSO-*d*_6_): δ 165.5, 163.1, 131.5, 115.6, 102.6, 50.4, 26.6, 24.1, 23.0, 22.5. HRMS calculated for C_10_H_13_NO_2_S (M+H) 212.0740, found 212.743.

#### Methyl 2-3-hydroxyadamantane-1-carboxamido)-4,5,6,7-tetrahydroben-zo[b]thiophene-3-carboxylate (AAT-015)

To a solution of 3-hydroxyadamantane-1-carbonyl chloride (108 mg, 0.503 mmol) in dry DCM (2 mL) was added dropwise a mixture of compound **3** (213 mg, 1.006 mmol) and triethylamine (70.1 μL, 0.503 mmol) in 0.8 mL of the same solvent. The reaction mixture was stirred at room temperature for 3 h, diluted with DCM and extracted with aq. NaHCO_3_ (saturated solution), aq. HCl (0.2 M) and brine. The organic layer was dried over magnesium sulfate and evaporated under reduced pressure. The crude product was purified by flash column chromatography on silica gel using hexane/ethyl acetate (6:4) as eluent to yield the title compound as a white solid (27 mg, 14%). ^1^H NMR (400 MHz, CDCl_3_): δ 11.6 (s, 1H), 3.9 (s, 3H), 2.7 (t, *J* = 5.6 Hz, 2H), 2.6 (t, *J* = 5.6 Hz, 2H), 2.4 (br. m, 2H), 1.9 (m, 6H), 1.8 (m, 8H), 1.6 (m, 2H). ^13^C NMR (100 MHz, CDCl_3_): δ 173.8, 167.4, 148.1, 130.7, 126.8, 111.4, 68.4, 51.5, 46.6, 44.2, 37.9, 34.9, 30.4, 26.3, 24.4, 23.0, 22.8. HRMS calculated for C_21_H_27_NO_4_S (M+H) 390.1734, found 390.1729.

#### 2-3-(hydroxyadamantane-1-carboxamido)-4,5,6,7-tetrahydrobenzo[b]thio-phene-3-carboxylic acid (4)

To a solution of **AAT-015** (50 mg, 0.128 mmol) in MeOH (5 mL) was added dropwise NaOH (0.385 mL, 0.385 mmol) and the reaction mixture was refluxed for 3 h. The reaction mixture was diluted with water and aq. HCl (1 M) was added until a pH of 2 was reached. The resulting precipitate was filtered and washed with ice water to give compound **4** (33 mg, 69%). ^1^H NMR (400 MHz, aceton-*d*_6_): δ 11.7 (s, 1H), 2.8 (t, *J* = 5.7 Hz, 2H), 2.6 (t, *J* = 5.7 Hz, 2H), 2.3 (br. m, 2H), 2.1 (s, 1H), 1.9 (m, 6H), 1.7 (m, 8H), 1.6 (m, 2H). ^13^C NMR (100 MHz, aceton-*d*_6_): δ 174.3, 168.2, 149.3, 131.8, 126.8, 112.1, 67.7, 47.4, 45.1, 38.7, 35.8, 31.2, 26.9, 24.8, 23.8, 23.5. HRMS calculated for C_20_H_25_NO_4_S (M+H) 376.1577, found 376.1573.

### *In vitro* binding assay

Membrane preparations originating from CHO-K1 cells stably transfected with either hCB_1_ or hCB_2_ (0.5 mg/tube, PerkinElmer, Massachusetts, US) and tritiated cannabinoid receptor agonist CP-55,940 (1.4 nM, CB_2_ agonist, Perkin Elmer, Boston, US) were used to determine inhibition constants of AAT-015 and AAT-778 in a competitive *in vitro* binding assay. A dilution series ranging from 1 pM to 1 mM in assay buffer (50 mM TRIS, 1 mM EDTA, 3 mM MgCl_2_ and 0.05% bovine serum albumin, pH adjusted to 7.4) was prepared for each ligand to be tested. Nonspecific binding was determined in the presence of AM251 (5 μM, CB_1_ inverse agonist, BIOTREND AG, Zürich, CH) and GW405833 (5 μM, CB_2_ partial agonist, Sigma Aldrich GmbH, Taufkirchen, DE), respectively. The samples were incubated at 30°C for 90 min and subsequently diluted with ice cold assay buffer (3 mL) in order to be vacuum-filtered through Whatman GF/C filters (pre-treated with aq. polyethylenimine, 0.05%). Each sample was washed twice with ice cold assay buffer (3 mL) and filtered again. After addition of scintillation cocktail (Ultima Gold, Perkin Elmer), radioactivity was measured using a Beckman LS 6500 Liquid Scintillation Counter. IC_50_ mean values were calculated after performing three independent experiments, each as triplicate. The Cheng-Prusoff equation (Cheng and Prusoff, 1973) was used to calculate the inhibition constants.

### Radiochemistry

#### Radiosynthesis of [^11^c]AAT-015

[^11^C]CO_2_ was produced by proton bombardment of nitrogen gas fortified with 0.5% oxygen in a Cyclone 18/9 cyclotron (18-MeV; IBA, Ottignies-Louvain-la-Neuve, Belgium) via the ^14^N(p,a)^11^C nuclear reaction. In a first step, nickel-based catalytic reduction of [^11^C]CO_2_ yielded [^11^C]methane which was subsequently iodinated to give [^11^C]MeI. In order to prepare [^11^C]AAT-015, the methylating agent was bubbled into a reaction mixture containing precursor **4** (1 mg) and cesium carbonate (5 mg) in DMF (0.6 mL) and stirred at 90°C for 3 min. After dilution of the crude product with water (1.4 mL), purification was performed by semi-preparative HPLC. The collected radiotracer was diluted with water (10 mL) and passed through a C18 cartridge (Waters, pre-conditioned with 5 mL EtOH and 5 mL water). After washing of the cartridge with water (5 mL), the product was eluted with EtOH (0.5 mL) into a sterile vial and diluted with water for injection (9.5 mL) to give a final formulation containing 5% of ethanol. The formulation was used for all *in vitro/in vivo* studies. For the purpose of quality control, an aliquot of the final formulation was injected into the analytical HPLC system. Identity of the product was confirmed by co-injection and comparison with the retention time of the standard reference. The specific activity was calculated by linear regression using a UV-intensity based calibration curve of standard reference AAT-015.

#### Radiosynthesis of [^11^C]AAT-778

[^11^C]AAT-778 was prepared according to the radiosynthetic procedure described in Section Radiosynthesis of [ 11 C]AAT-015, starting from precursor **2**.

### *In vitro* autoradiography

Autoradiography was performed on rat/mouse spleen tissue. Sections were prepared in 20 μm thickness using a cryostat (Cryo-Star NX 50; Thermo Scientific). The tissue slices were adsorbed to SuperFrost Plus (Menzel) slides and stored at −20°C until use. After thawing on ice for 10 min, sections were pre-incubated in the assay buffer containing 50 mM TRIS, 5 mM MgCl_2_, 2.5 mM EDTA, and 0.5% BSA (pH 7.4). Slices were dried and incubated in a humidified chamber with [^11^C]AAT-015 (5 nM) or [^11^C]AAT-778 (5 nM) in assay buffer for 15 min at room temperature. In order to test for specificity toward CB_2_, a solution containing both radiotracer and an excess of CB_2_ specific partial agonist GW405833 (10 μM) was prepared and added to the tissue. The tissue slices were washed twice with assay buffer (each 2 min) and twice with distilled water (each 5 s) on ice, air dried, and exposed to a phosphor imager plate for a period of 25 min. The plate was scanned using a BAS5000 reader (Fujifilm, Dielsdorf, CH).

### *In vivo* PET

PET and CT scans were obtained with a Super Argus PET/CT tomograph (Sedecal, Madrid, Spain) after injection of [^11^C]AAT-015 (10–15 MBq, 0.02–0.10 nmol) into the tail of male Wistar rats (416 ± 32 g, *n* = 4) immobilized by isoflurane. Under baseline conditions, radiotracer accumulation was recorded in the region of the spleen in dynamic PET acquisition mode over 60 min (*n* = 2). During this whole time period rats were kept under anesthesia, body temperature and respiratory rate were constantly monitored. Under blockade conditions, 1.5 mg/kg GW405833 was injected shortly before radiotracer application in two of the four rats. Acquired PET data were reconstructed as user-defined time frames with a voxel size of 0.3875 × 0.3875 × 0.775 mm. For anatomical orientation, CT scans were acquired after each PET scan. Images were evaluated with PMOD v3.4 (PMOD Technologies Inc., Zurich, CH) software. Regions of interest (spleen, liver, and muscle) were drawn manually using the PMOD fusion tool. Time activity curves (TACs) for spleen, liver and muscle were expressed as standardized uptake values (SUV) which are the decay-corrected radioactivity per cm^3^ divided by the injected radioactivity dose per gram of body weight.

## Results

### Chemistry and radiochemistry

**Scheme 1:**
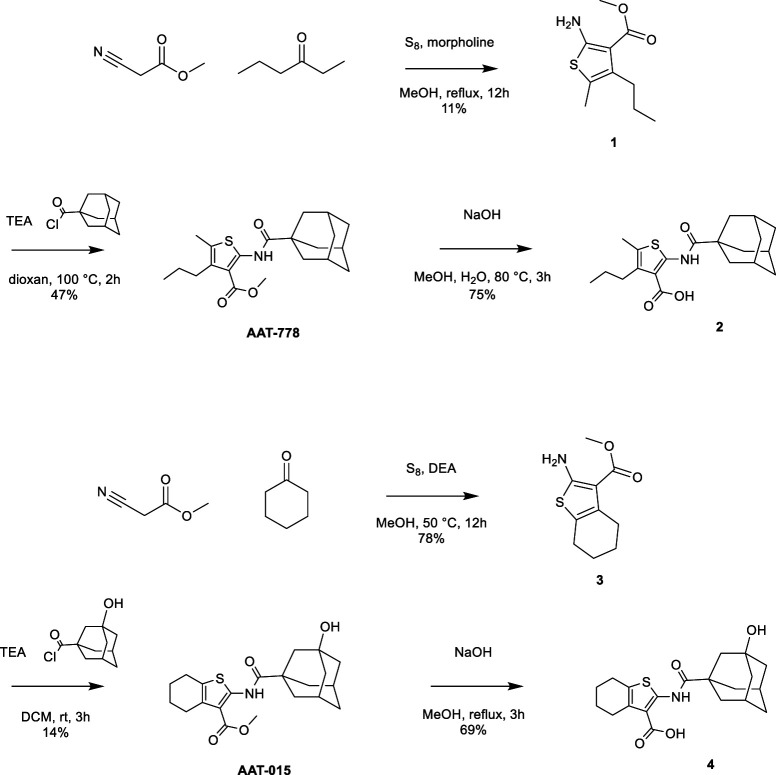
**Synthesis of thiophene-based CB_2_ ligands AAT-778 and AAT-015 and their respective precursors for C-11 radiolabeling**.

Thiophene-based reference compounds AAT-778 and AAT-015 were synthesized starting from commercially available methyl cyanoacetate and the appropriate ketones as depicted in Scheme 1. The thiophene amine compounds **1** and **3** were obtained in 11 and 78%, respectively under the Gewald reaction conditions (Puterová et al., 2010). Subsequent coupling of **1** and **3** with 1-adamantanecarbonyl chloride and 3-hydroxyadamantane-1-carbonyl chloride, respectively, yielded the reference compounds AAT-778 and AAT-015. Saponification of the ester moiety of AAT-778 afforded precursor **2** for radiolabeling in 75% yield. Similarly, compound **4** was obtained from AAT-015 in 69% yield.

**Scheme 2:**
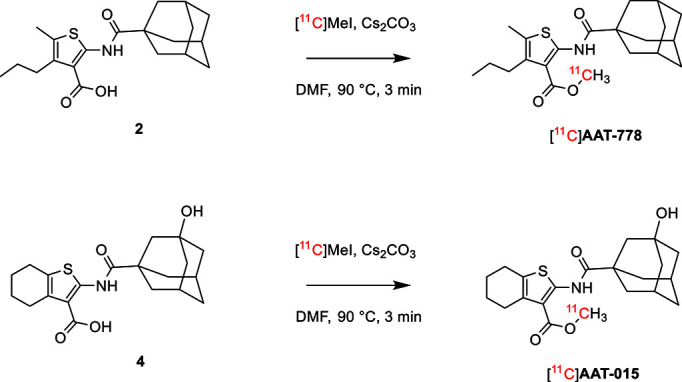
**Radiosynthesis of [^11^C]AAT-778 and [^11^C]AAT-015**.

**Figure 1:**
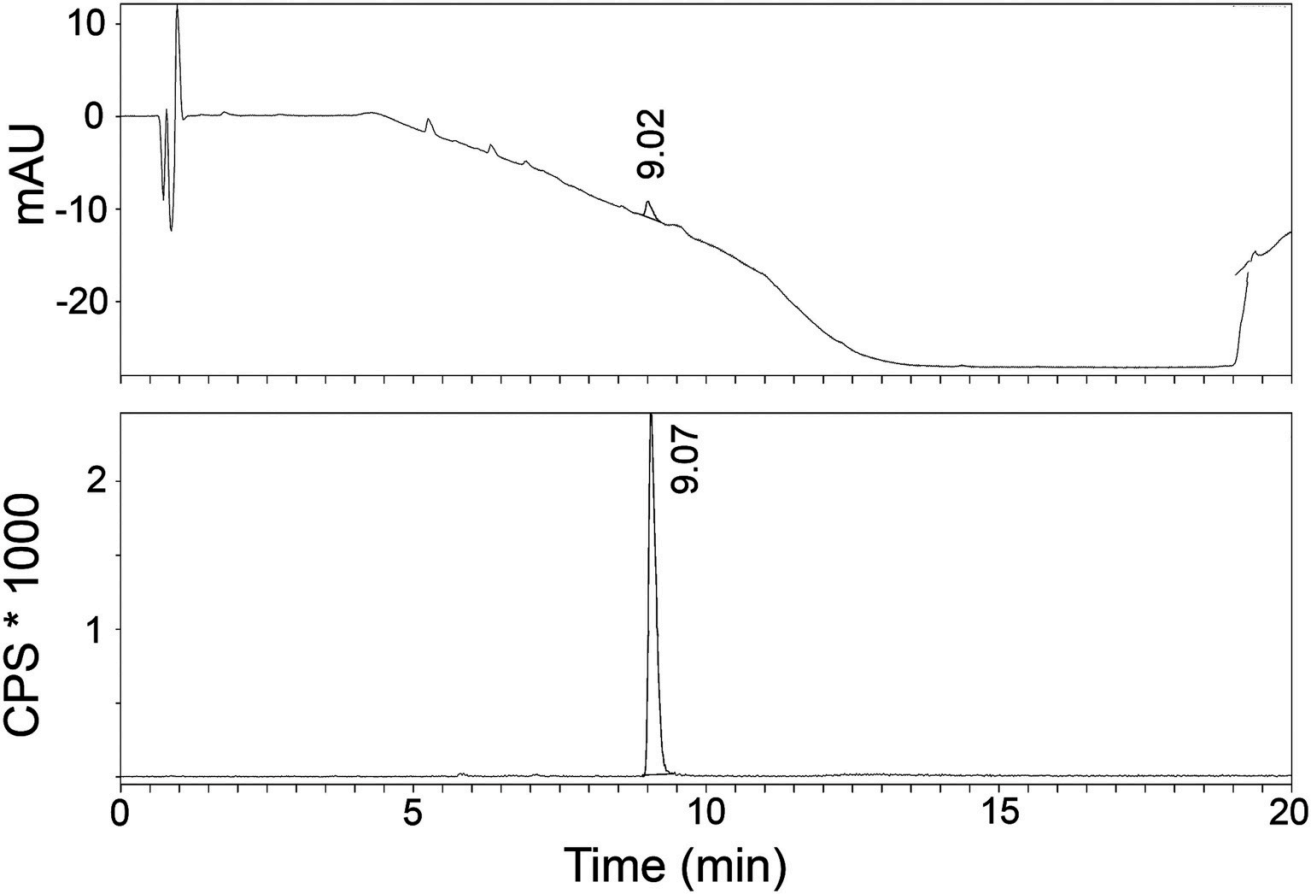
**A typical analytical HPLC profile of [^11^C]AAT-015 [UV-trace (top) and radio-trace (bottom)]**.

**Figure 2:**
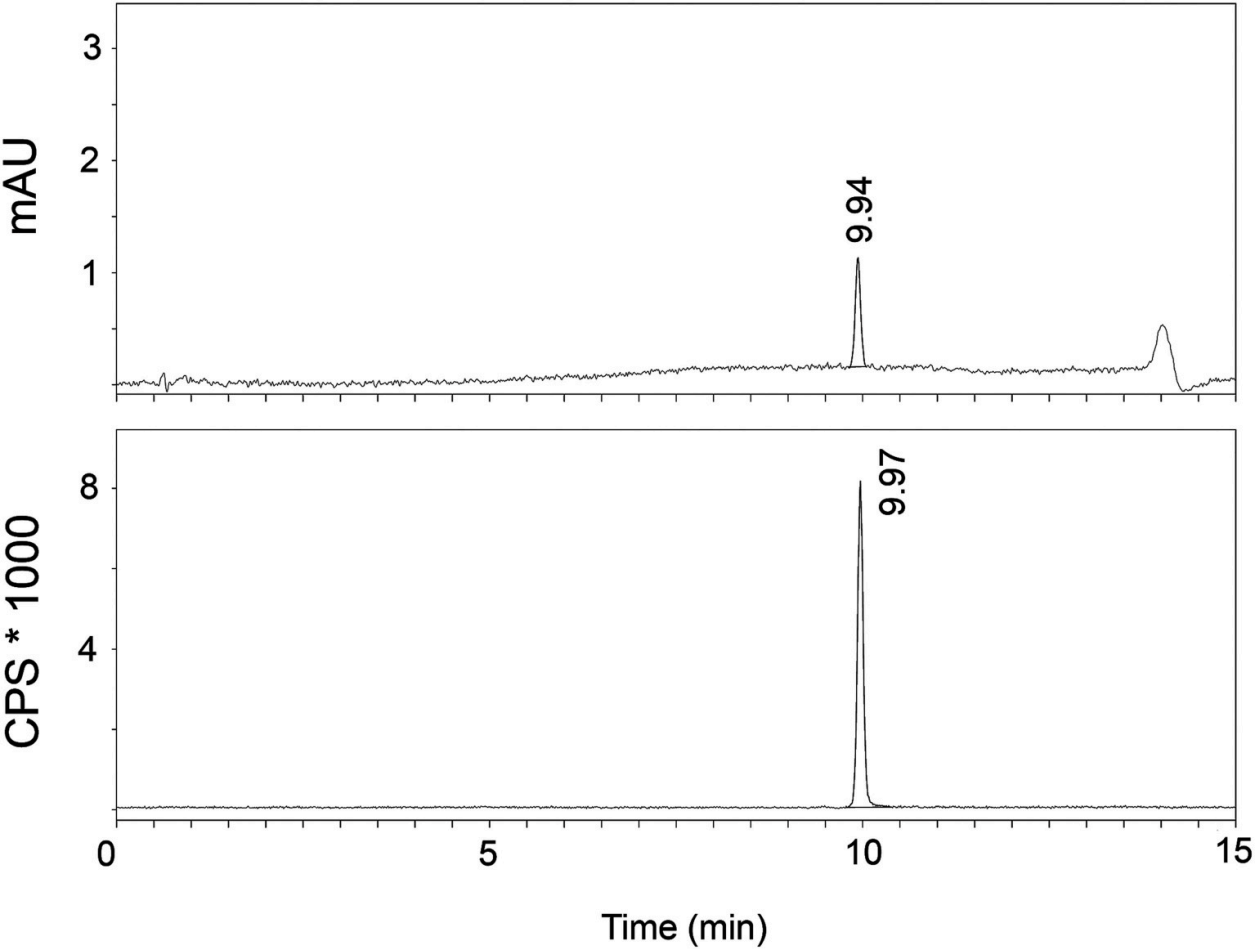
**A typical analytical HPLC profile of [^11^C]AAT-778 [UV-trace (top) and radio-trace (bottom)]**.

The radiosynthesis of [^11^C]AAT-778 and [^11^C]AAT-015 was accomplished by methyl ester formation from their corresponding carboxylic acids with [^11^C]MeI (Scheme 2). The obtained radiochemical yields ranged from 12 to 37% (decay corrected) with specific activities between 96 and 449 GBq/μmol at the end of synthesis. In all cases, a radiochemical purity of 99% was attained after semi-preparative HPLC purification (Figures 1, 2). The total radiosynthesis time from end of bombardment to the end of synthesis was 40 min.

### *In vitro* characterization

Binding affinity studies were performed using membranes stably transfected with human CB_1_ and CB_2_, respectively. A K_i_-value of 4.3 ± 0.7 nM toward CB_2_ and a 250-fold selectivity over CB_1_ (Table 1) was obtained for compound AAT-778. We found that introduction of a polar hydroxyl on the adamantane moiety and ring closure to form the rather rigid cyclohexene did not impact the binding affinity and selectivity toward CB_2_ as shown by the K_i_-value of 3.3 ± 0.5 nM (toward CB_2_) and 300-fold selectivity over CB_1_ for AAT-015. One limitation of our *in vitro* work is that besides CB_1_ we did not investigate the binding affinity of our ligands to other targets. Furthermore, clogP values depicted in Table 1 indicate a significant reduction of lipophilicity after introduction of the hydroxyl group on the adamantane moiety in AAT-015.

**Table 1:** **K_i_-values of known CB_1_/CB_2_ ligands (entry 1–3; Showalter et al., 1996; Lan et al., 1999; Valenzano et al., 2005) and newly synthesized compounds (entry 4, 5)**.

	**K_i_ (hCB_2_) [nM]**	**K_i_ (hCB_1_) [nM]**	**clogP**
1. CP-55940	0.7 ± 0.02	0.6 ± 0.1	5.82
2. AM251	2290 ± 900	7.5 ± 1.1	5.62
3. GW405833	3.9 ± 1.6	4772 ± 1676	6.90
4. AAT-778	4.3 ± 0.7	1100 ± 100	6.00
5. AAT-015	3.3 ± 0.5	1000 ± 200	4.11

**Figure 3:**
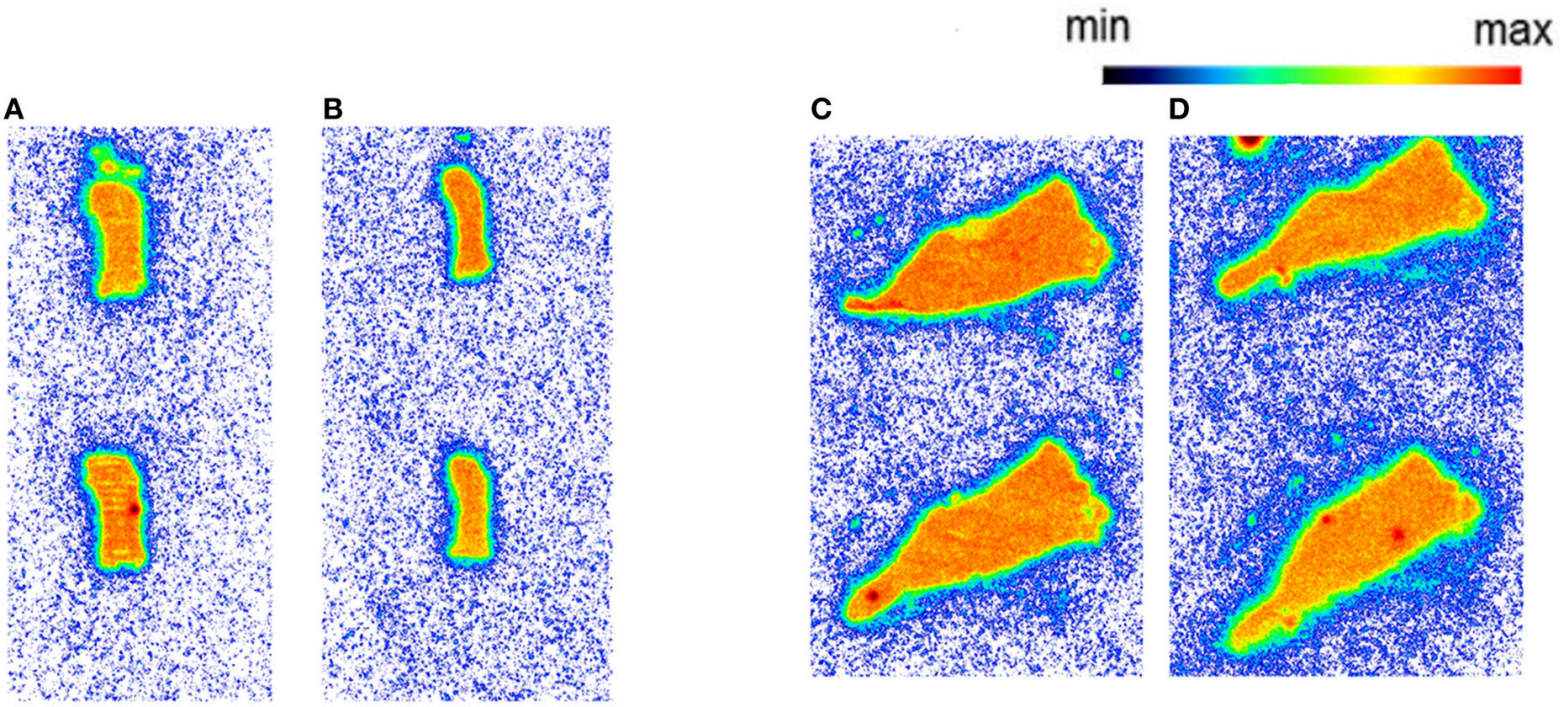
**The autoradiography results obtained with 5 nM [^11^C]AAT-778 using spleen tissue sections. (A)** Mouse spleen under baseline condition; **(B)** Mouse spleen under blockade conditions (10 μM GW405833); **(C)** Rat spleen under baseline conditions; **(D)** Rat spleen under blockade conditions (10 μM GW405833); *n* = 2.

**Figure 4:**
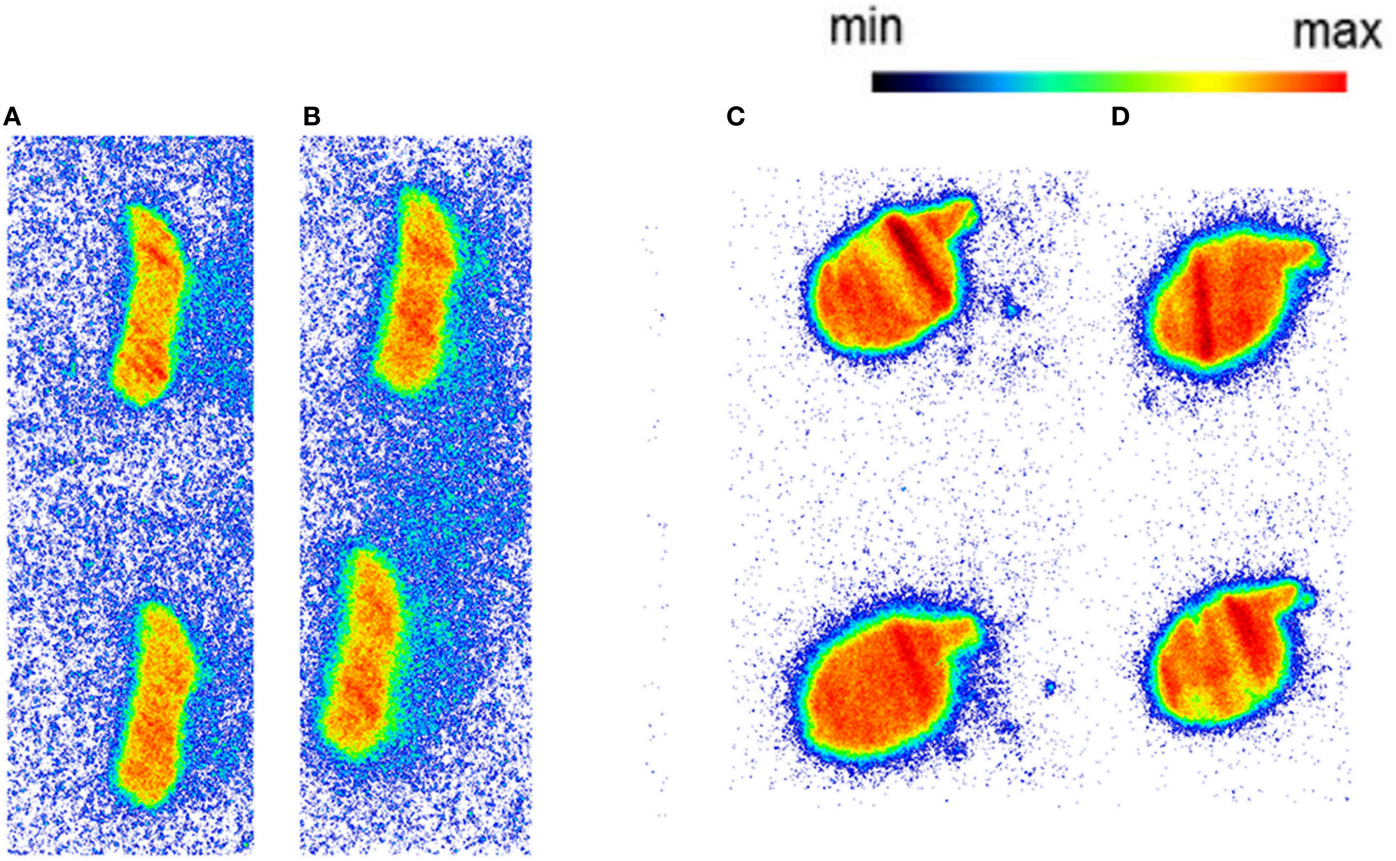
**The autoradiography results obtained with 5 nM [^11^C]AAT-015 using spleen tissue sections**. **(A)** Mouse spleen under baseline condition; **(B)** Mouse spleen under blockade conditions (10 μM GW405833); **(C)** Rat spleen under baseline conditions; **(D)** Rat spleen under blockade conditions (10 μM GW405833); *n* = 2.

Autoradiography with CD1 mouse and Wistar rat spleen tissue revealed binding of [^11^C]AAT-778 to the spleen tissue in both species under baseline conditions (Figures 3A,C). No blocking effect was observed using an excess of CB_2_-specific ligand GW405833, suggesting that the binding was unspecific (Figures 3B,D). Similar results were obtained with the less lipophilic [^11^C]AAT-015 (Figure 4).

### *In vivo* characterization

**Figure 5:**
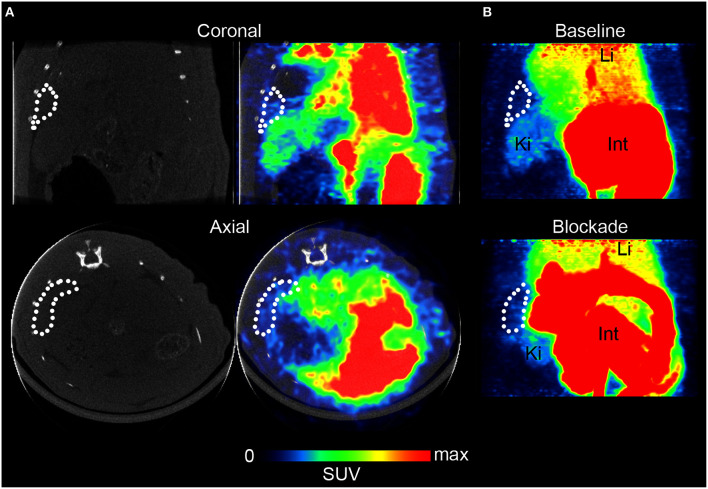
**Rat spleen PET/CT images after injection of [^11^C]AAT-015 (averaged 0–60 min post injection). (A)** Coronal and axial slices of the abdominal region. Spleen is encircled in the CT and PET/CT images, demonstrating low uptake of [^11^C]AAT-015. SUV max = 4. **(B)** Maximal intensity projection (MIP) images of the abdominal region under baseline (same rat as in **A**) and blockade conditions (injection of 1.5 mg/kg GW405833 shortly before radiotracer). Spleen is encircled. Li, liver; Ki, kidney; Int, intestine. SUV max = 6.

For *in vivo* evaluation, [^11^C]AAT-015 was selected due to the favored lipophilicity profile. Coronal and axial PET/CT slices of the abdominal region of a representative Wistar rat which was administered with [^11^C]AAT-015 are shown in Figure 5. The spleen is encircled in all the images depicted in Figure 5 and shows only low uptake of [^11^C]AAT-015.

**Figure 6:**
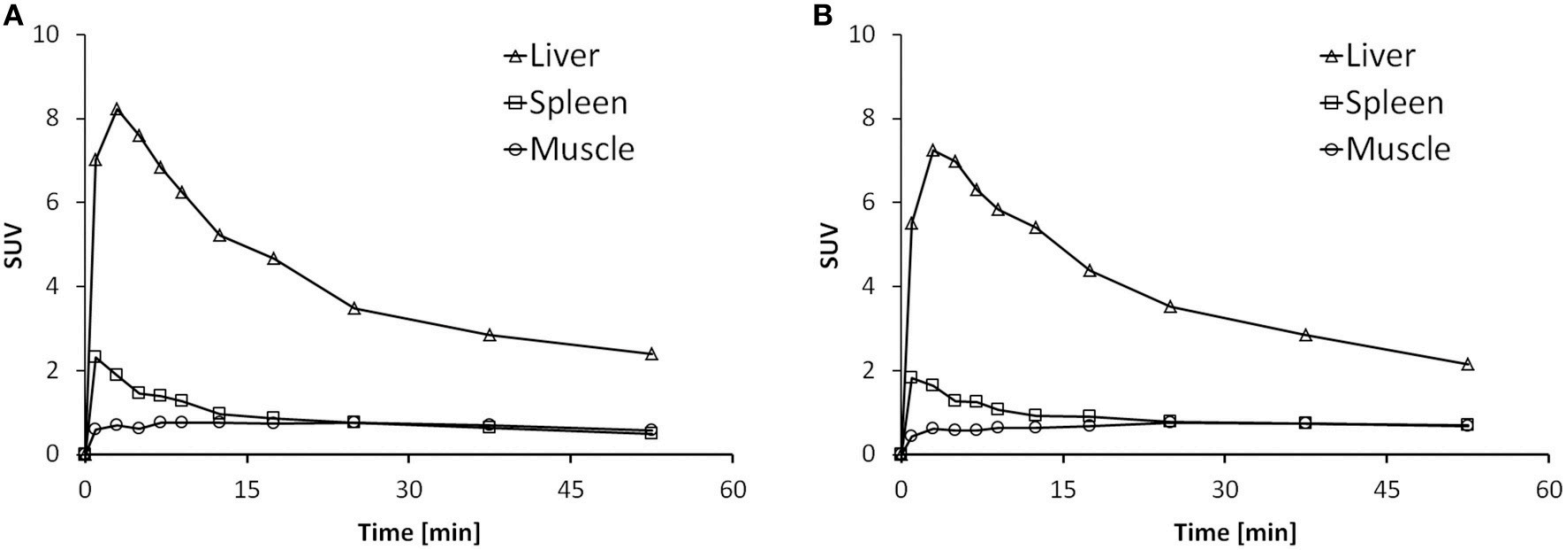
**Representative time activity curves (TACs) showing the standardized uptake values (SUV) of spleen, liver, and muscle in the time course of 60 min after injection of [^11^C]AAT-015 into Wistar rats. (A)** Injection of tracer only; **(B)** Injection of tracer and 1.5 mg/kg GW405833 (shortly before tracer).

Time activity curves (TACs) are depicted in Figure 6 for the liver, spleen and muscle under baseline (Figure 6A) and blockade (Figure 6B) conditions. There was no difference in the spleen uptake of [^11^C]AAT-015 when comparing the TACs under baseline and blockade conditions. Radioactivity washout from the spleen was rather rapid, suggesting a perfusion-like profile rather than specific accumulation in the target organ. Thus, the uptake in the spleen was not specific.

## Discussion

Thiophene-based structures with high affinity toward CB_2_ have been previously reported by Nelson et al. (2012). A series of derivatives were screened within the context of pain therapy. We developed two novel thiophene-based compounds, with an ester functionality which allows generating the precursors for C-11 radiolabeling. The designated compounds were obtained in a two-step synthetic approach involving Gewald reaction to form the thiophene amine. In the Gewald reaction, methyl cyanoacetate was treated with the corresponding ketones, followed by reaction with sulfur to afford thiophene amine derivatives **1** and **3**. In the case of the linear 3-hexanone, two different regioisomers were obtained which were not separable by column chromatography. Therefore, an additional recrystallization step was necessary in order to obtain pure compound **1** which may partly explain the lower yield when compared to the synthesis of 2-aminothiophene **3**. Coupling of the amine building blocks to adamantanecarboxylic acid using HBTU as the coupling reagent did not give the desired amides. However, amides AAT-015 and AAT-778 could be obtained by the reaction of the acid chloride with the corresponding thiophene amines. Subsequently, saponification of the ester intermediates gave the acid precursors in good chemical yields.

Binding affinity studies revealed that the introduction of the hydrophilic OH-group at the adamantane moiety did not have any influence on the affinity toward the CB_2_ receptor since similar K_i_-values were obtained for both AAT-015 and AAT-0778. Furthermore, the introduction of rigidity by ring-closure did not affect the binding affinity, which enlarges the compound selection and simplifies compound synthesis. As expected, the lipophilicity of AAT-015 was significantly reduced to 4.11 compared to 6.00 for AAT-778, which is highly desired. Carbon-11 radiolabelings of the acid precursors were carried out under similar conditions for both tracers and yielded comparable results in terms of radiochemical yield and purity as well as specific activity. In autoradiography studies with rodent spleen tissue [^11^C]AAT-778 exhibited high unspecific binding. We speculate that this high unspecific binding is related to the high lipophilicity of the molecule. In order to reduce this unspecific binding, we modified AAT-778 by introducing a hydroxyl moiety on the adamantine ring to deliver AAT-015. However, despite the significant reduction of clogP, [^11^C]AAT-015 still revealed a high unspecific binding in mouse and rat spleen tissues. PET studies of [^11^C]AAT-015 confirmed the lack of specificity toward CB_2_-positive spleen tissue *in vivo*. Possible reasons for the described *in vivo* results might include high plasma protein binding and rapid metabolism. The ester functionality can be cleaved by the action of carboxylesterases which are present in the plasma. This would, however, lead to a loss of the radiolabel from the core structure.

In conclusion, the novel thiophene-based radiotracers [^11^C]AAT-778 and [^11^C]AAT-015 are not suitable CB_2_ PET tracers due to the lack of specificity for CB_2_-positive spleen tissue and therefore were not further evaluated in neuroinflammatory animal models. Nonetheless, the high *in vitro* binding affinity toward CB_2_ as well as the high selectivity over CB_1_ makes this class of compounds interesting for further structural optimization toward improved physicochemical and pharmacological properties.

## Author contributions

AH performed the organic synthesis of precursor **4** and reference compound AAT-015 and conducted the IC_50_ studies with AAT-015_._ He further performed the radiosynthesis and autoradiography studies with [^11^C]AAT-015 and wrote the manuscript. AM planned and evaluated the *in vivo* experiments and helped writing the manuscript. RS performed the radiosynthesis and autoradiography with [^11^C]AAT-778. MW was involved in result discussions and revised the manuscript. CM and AL synthesized precursor **2** and reference compound AAT-778 and determined the Ki-value of AAT-778. RS revised and approved the manuscript. LM and SM planned and organized the whole project and approved the manuscript.

### Conflict of interest statement

The authors declare that the research was conducted in the absence of any commercial or financial relationships that could be construed as a potential conflict of interest.
